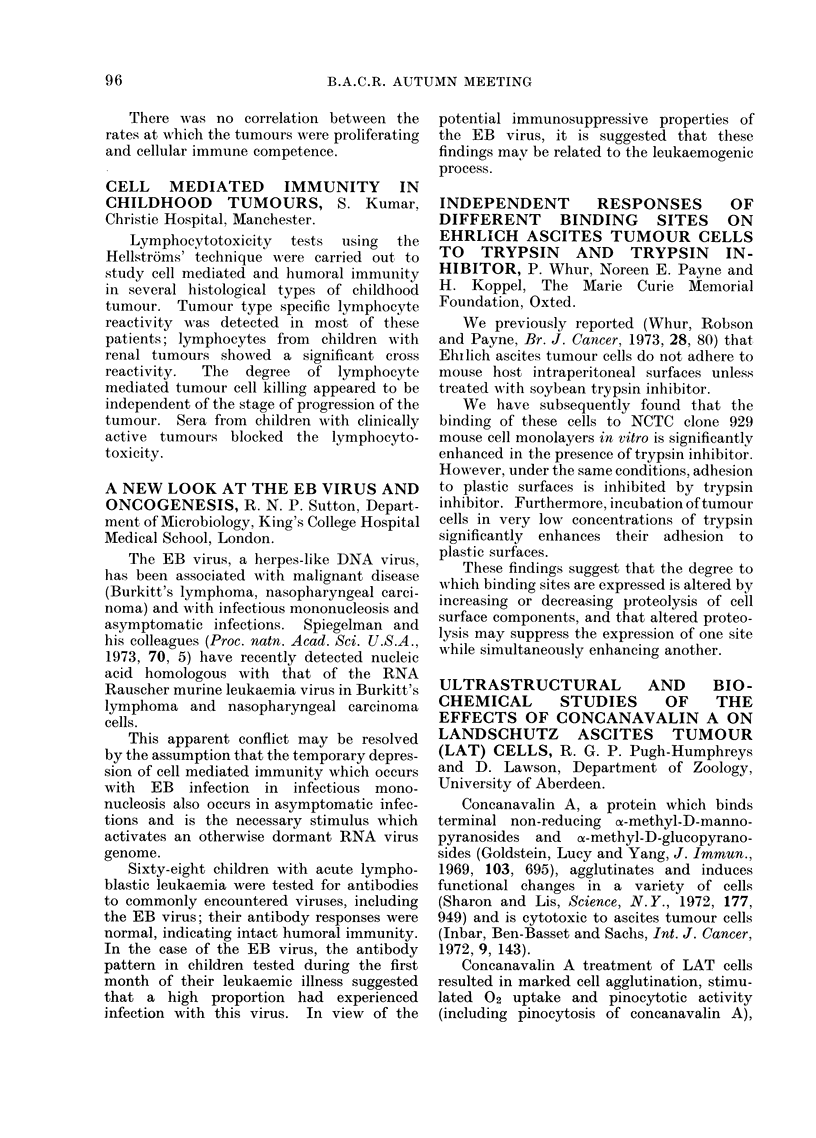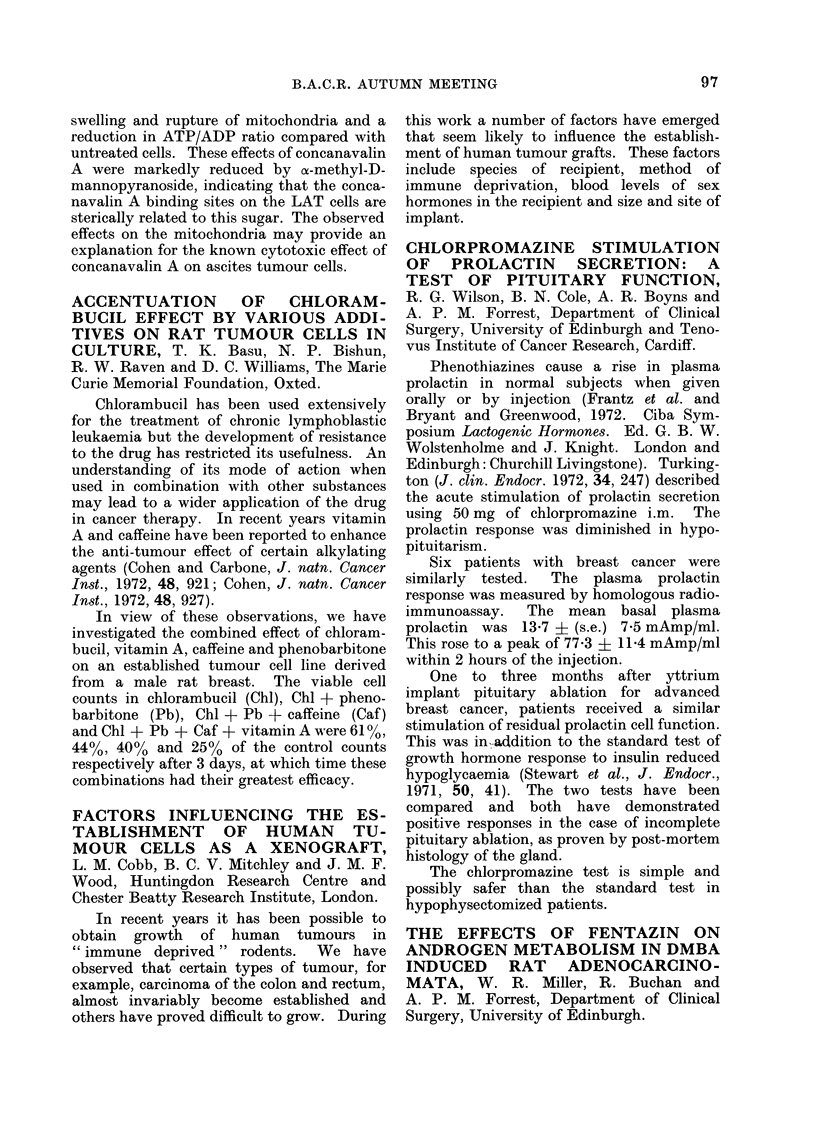# Proceedings: Ultrastructural and biochemical studies of the effects of concanavalin A on Landschutz ascites tumour (LAT) cells.

**DOI:** 10.1038/bjc.1974.29

**Published:** 1974-01

**Authors:** R. G. Pugh-Humphreys, D. Lawson


**ULTRASTRUCTURAL AND BIOCHEMICAL STUDIES OF THE EFFECTS OF CONCANAVALIN A ON LANDSCHUTZ ASCITES TUMOUR (LAT) CELLS,** R. G. P. Pugh-Humphreys and D. Lawson, Department of Zoology, University of Aberdeen.

Concanavalin A, a protein which binds terminal non-reducing α-methyl-D-mannopyranosides and α-methyl-D-glucopyranosides (Goldstein, Lucy and Yang, *J. Immun.*, 1969, **103**, 695), agglutinates and induces functional changes in a variety of cells (Sharon and Lis, *Science, N.Y.*, 1972, **177**, 949) and is cytotoxic to ascites tumour cells (Inbar, Ben-Basset and Sachs, *Int. J. Cancer*, 1972, **9**, 143).

Concanavalin A treatment of LAT cells resulted in marked cell agglutination, stimulated $O_2$ uptake and pinocytotic activity (including pinocytosis of concanavalin A), swelling and rupture of mitochondria and a reduction in ATP/ADP ratio compared with untreated cells. These effects of concanavalin A were markedly reduced by α-methyl-D-mannopyranoside, indicating that the concanavalin A binding sites on the LAT cells are sterically related to this sugar. The observed effects on the mitochondria may provide an explanation for the known cytotoxic effect of concanavalin A on ascites tumour cells.